# Exposure of Macrophages to Low-Dose Gadolinium-Based Contrast Medium: Impact on Oxidative Stress and Cytokines Production

**DOI:** 10.1155/2018/3535769

**Published:** 2018-12-02

**Authors:** Te-I Weng, Huang Jen Chen, Chen-Wen Lu, Yu-Chin Ho, Jia-Lun Wu, Shing-Hwa Liu, Jong-Kai Hsiao

**Affiliations:** ^1^Forensic and Clinical Toxicology Center, National Taiwan University College of Medicine and National Taiwan University Hospital, Taipei, Taiwan; ^2^Department of Forensic Medicine, College of Medicine, National Taiwan University, Taipei, Taiwan; ^3^Department of Emergency Medicine, National Taiwan University Hospital, Taipei, Taiwan; ^4^Institute of Toxicology, College of Medicine, National Taiwan University, Taipei, Taiwan; ^5^Department of Medical Imaging, Taipei Tzu Chi Hospital, Buddhist Tzu-Chi Medical Foundation, New Taipei City, Taiwan; ^6^School of Medicine, Tzu Chi University, Hualien, Taiwan

## Abstract

The toxicity of gadolinium-based contrast agents (GBCAs) has drawn a lot of attention. Nephrogenic systemic fibrosis (NSF), a lethal disease related to the use of GBCAs, is still not understood. Recently, gadolinium retention is found in brain tissues after repeated use of GBCAs in magnetic resonance imaging (MRI). However, most of the works investigating the toxicity of GBCAs are focusing on its high-concentration (0.5–10 mM) part, which is not reflective of the physiological conditions in human beings. Macrophages play a regulatory role in immune responses and are responsible for the fibrosis process. Their role in gadolinium retention and the pathogenesis of NSF, however, has seldom been investigated. This study aimed to evaluate the immune response generated by macrophages (RAW 264.7) exposing to low levels of GBCAs. The incubation concentration of GBCAs, including Omniscan®, Primovist®, Magnevist®, and Gadovist®, is proportional to the level of gadolinium uptake when detected via inductively coupled plasma mass spectrometry (ICP-MS) and imaged by MRI, whereas Primovist® treatment groups have highest gadolinium uptake among all of the tested concentrations. Low-concentration (2.5 *μ*mol/L) Gd chloride or GBCAs exposure promoted the reactive production of oxygen species (ROS), nitrate/nitrite, prostaglandin E2 (PGE2), and suppressed the potential of mitochondrial membrane. There was higher ROS, nitrate/nitrite, and PGE2 production in the Primovist®, Omniscan®, and Magnevist® groups compared to the Gadovist® group. In face of lipopolysaccharide (LPS) stimulation, Primovist®, Omniscan®, and Magnevist® groups exhibited elevated nitrite/nitrate and suppressed IL-1*β* secretion and IL-6 and IL-10 secretion. Moreover, upon LPS stimulation, there is decreased TNF-*α* secretion 4 hours after Primovist® or Omiscan® exposure but the TNF-*α* secretion increased at 24 hours. Our data suggest that there is upregulated inflammation even in the presence of low levels of GBCAs, even similar to the physiological condition in murine macrophage. Further investigation of GBCAs on the human macrophage or *in vivo* animal study may clarify the role of macrophage on the pathogenesis of NSF and other GBCAs-related disease.

## 1. Introduction

Gadolinium-based contrast agents (GBCAs) have been used clinically in magnetic resonance imaging (MRI) to detect malignancy. It is also used to verify vascular abnormality and tissue perfusion defects in stroke and myocardial infarction [1]. However, safety concerns were recently brought up that nephrogenic systemic fibrosis may be caused by repeated use of GBCAs [2]. Several studies on the pathogenesis of GBCAs-related NSF have proposed that impaired clearance of gadolinium by kidneys could lead to tissue accumulation of dissociated gadolinium (Gd) [3]. Recently, gadolinium deposition in brain tissues has been observed in patients and animals with normal renal function when receiving repeated MRI along with GBCAs administration [4–6]. However, the cause of gadolinium retention in normal tissues and its potential hazards, as well as its role in the process of NSF, remain unknown.

Studies have demonstrated a variety of adverse effects associated with GBCAs administration. Histopathological and molecular evidences showed obvious damage in the spleen, lungs, and renal tissues [7]. GBCAs were found to induce higher cytotoxicity in a confluent proximal tubular epithelial cell line when compared with iodinated contrast agents [8]. Moreover, ionic gadolinium dissociated from gadolinium chloride can cause in vitro neurotoxicity [9]. Because of the widespread use of chelated gadolinium in the clinical field, the toxicity of chelated and ionic forms of gadolinium calls for more thorough investigation.

Macrophages play an important role in the regulation of immune and inflammatory responses. When activated, macrophages secrete a variety of cytokines, including TNF-*α*, interleukin (IL)-1 beta, IL-6, IL-10, nitric oxide (NO), and eicosanoids such as prostaglandin E2 (PGE2) [10, 11]. These substances contribute to tissue damage mediated by activated macrophages. Moreover, it has been demonstrated that activated macrophages can contribute to early events in various fibrotic processes [12]. These results suggest that macrophages could play a pivotal role in NSF pathogenesis.

The cytotoxicity of GBCAs has been studied in macrophages [13, 14]. Most studies, however, focused on the responses produced by high concentrations (0.5–10 mM) of GBCAs, which may result in the production of IL-1*β* or iNOS [13, 14]. Human studies have revealed maximum plasma gadolinium concentrations of 65.7 *μ*g/mL. The plasma clearance of GBCAs is 1.1–2.15 ml/min/kg. Moreover, the elimination half-life of GBCAs is 1.2 h (1.0–1.8 h) for persons with normal renal function, which indicates that very low concentrations and trace amounts of GBCAs can also interact with macrophages [15]. Previous studies focusing on changes due to high gadolinium concentrations (0.5–10 mM) may not be directly applicable to the pathogenesis of NSF or gadolinium deposition in the brain [13, 14]. As a result, the present study tends to examine the oxidative and immune effects of GBCAs at the concentration level lower than the human serum level when administered intravenously.

## 2. Material and Methods

### 2.1. Chemicals

Gadolinium (as GdCl3·xH2O 99% purity) was obtained from Sigma-Aldrich. Primovist® (gadoxetic acid; Gd-EOB-DTPP; 500 mM/mL; human dose, 25 *μ*mol/kg), Magnevist® (gadopentetic acid; 500 mM/mL; human dose, 100–300 *μ*mol/kg), and Gadovist® (gadobutrol; 1000 mM/mL; human dose, 100–300 *μ*mol/kg) were purchased from Bayer Schering Pharma or Bayer HealthCare Pharmaceuticals. Omniscan® (gadodiamide; 500 mM/mL; human dose, 100 *μ*mol/kg) was purchased from GE Healthcare Inc.

### 2.2. Cell Cultures

RAW 264.7, a murine macrophage cell line, was purchased from Culture Collection and Research Centre, Hsin-Chu, Taiwan. The cells were cultured in DMEM medium (Gibco, Grand Island, NY, USA) supplemented with 2 mM glutamine, antibiotics (100 U/mL penicillin A and 100 U/mL streptomycin), and 5% heat-inactivated fetal bovine serum (Gibco). Also the cells were maintained in a 37°C humidified incubator containing 5% CO2. The cells were passaged when they reached 70%–80% confluence.

### 2.3. Treatment of RAW 264.7 with GBCAs

Equal number (8 × 10^5^) of RAW 264.7 was plated in 60 mm plastic culture dishes. The cells were exposed to different concentrations of Gd chloride or GBCAs (Primovist®, Omniscan®, Magnevist®, or Gadovist®) at concentrations of 0.25 *μ*M to 2.5 *μ*M.

### 2.4. Cell Viability Assay

Cell viability was evaluated by MTT (3-[4, 5-dimethylthiazol-2-yl]-2,5-diphenyltetrazolium bromide) assay. The Gd chloride- and GBCAs-treated cells were grown in triplicate in 24-well plates for 24 h. Later, MTT was added to the medium at a final concentration of 0.5 mg/mL, and the cells were incubated for one hour at 37°C in 5% CO_2_. After incubation, when the dark-blue formazan dye generated by the living cells became proportional to the number of live cells, the absorbance was measured at 570 nm using a microplate reader. MTT data were shown as the percentage of the average values of the control cells.

### 2.5. Cell Morphology

The RAW 264.7 cells were seeded on 10 cm plates with fresh medium and exposed to 2.5 *μ*M GBCAs for 24 h. At the end of the treatment, the cells were washed twice with PBS and visualized using an inverted microscope (BX51, Olympus, Japan) with 200× magnification.

### 2.6. MRI

MRI was performed using a clinical 3.0 T MR System (Signa Excite; GE Healthcare Bio-Science, Piscataway, NJ, USA) as described previously [16]. Briefly, the cell samples were centrifuged and placed in a water tank, which was placed in an 8-channel head coil. Two-dimensional T1-weighted fast spin-echo pulse sequences were used (TR/TE = 550/13 ms). The slice thickness was 1.0 mm, with a 0.5 mm gap, and the field view was 14 cm × 10 cm with a matrix size of 288 × 192. The scan time was 4 min and 5s with a repetition of 2. These images were further analyzed at a workstation provided by GE Healthcare (Advantage workstation 4.2) with the free Image J software. We measured the signal intensity of each cell pallets to obtain quantitative data, which is also an indirect method to determine the gadolinium deposition in the cells. The gross phenomenon of cells via MRI indirectly proved the interactions between macrophages and GBCAs [16].

### 2.7. Reactive Oxygen Species (ROS) Measurements

The production of ROS under oxidative stress was measured using the OxiSelect™ Intracellular ROS Assay Kit (Cell Biolabs, San Diego, CA, USA). Cells were cultured in 96-well plates after the treatment of 2.5 *μ*M GBCAs for 4 or 24 h then loaded with 1 mM of the cell-permeable fluorogenic probe 2',7'-dichlorodihydrofluorescin diacetate (DCF-DA) for 1 h. In brief, the DCF-DA was finally oxidized to high fluorescent 2',7'-dichlorodihydrofluorescin by intracellular ROS. The fluorescence intensity was measured using a fluorescence plate reader (480 nm/530 nm) [17].

### 2.8. Mitochondrial Membrane Potential Measurements

Alterations in the mitochondrial membrane potential were analyzed using the tetramethylrhodamine ethyl ester (TMRE) mitochondrial membrane potential assay (Cayman Chemical, Ann Arbor, MI). The methods were modified from those described in a previous study [18]. Cells were cultured in 96-well plates after the treatment of 2.5 *μ*M GBCAs for 4 or 24 h. Treated cells were incubated with 5–500 nM TMRE in a serum-free medium at 37°C for 30 min. Active mitochondria absorb positively charged TMRE due to its negative charge. Depolarized or inactivated macrophages have low membrane potentials and fail to absorb TMRE. We used a fluorescence plate reader with excitation at 530 nm and emission at 580 nm to analyze the accumulation of TMRE. Changes in fluorescence were calculated following the manufacturer's instructions.

### 2.9. Measurements of Cytokines, Nitrite/Nitrate, and Prostaglandin E2 (PGE2) Levels

Enzyme-linked immunosorbent assay kits were used to measure the levels of interleukin (IL)-1*β*, IL-6 (R&D Systems), tumor necrosis factor (TNF)-*α* (Assaypro), IL-10 (LEGEND MAX, BioLegend), and PGE2 (Cayman Chemical, Ann Arbor, MI) in supernatants from macrophages exposed to the Gd^3+^ compounds for 4 or 24 h. Concentrations of nitrite were determined by a nitrate/nitrite colorimetric assay kit (R&D Systems). In LPS stimulation experiments, cells were stimulated with LPS for 4 h or 24 h following overnight Gd chloride or GBCAs incubation.

### 2.10. Statistics

Data are presented as means ± standard error (SEM). Statistical analysis was performed using one-way analysis of variance followed by the Dunnett test for each paired experiment. *p* < 0.05 was considered as statistically significant.

## 3. Results

### 3.1. Effects of Different Kinds of GBCAs in Mouse's Macrophage RAW Cells

**Figure 1:**
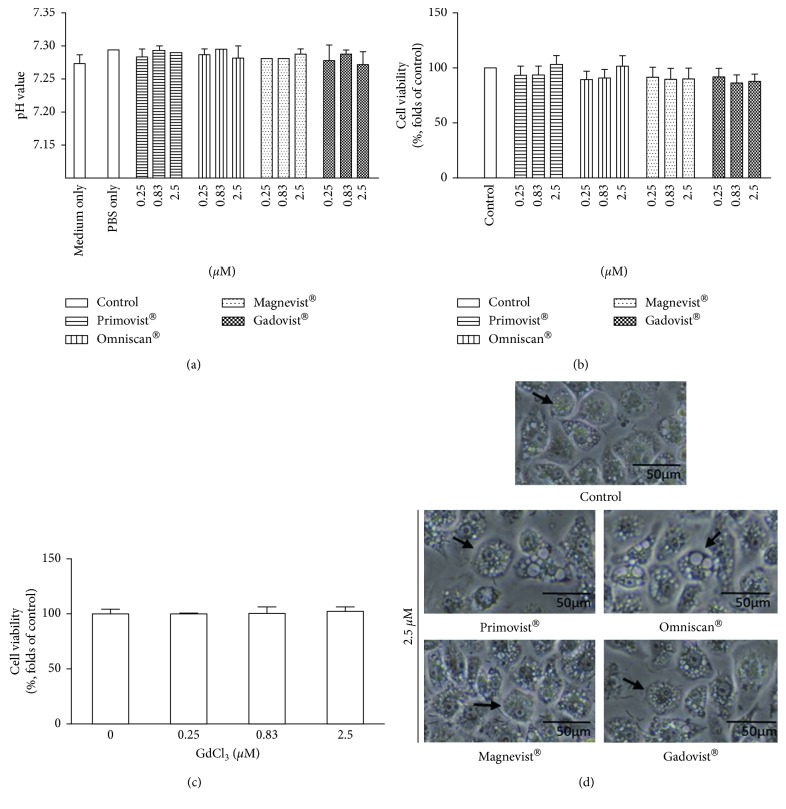
Cell viability of different types of gadolinium-based contrast agents (GBCAs) and gadolinium chloride on RAW 264.7 cells. Macrophages (RAW 264.7) were incubated in the absence or presence of 0.25, 0.83, and 2.5 *µ*M/mL of various GBCAs or Gd chloride for 24 h. The pH values of PBS diluted GBCAs were checked (a) (*n* = 3). The effect of GBCAs or Gd chloride on cell viability was determined by MTT assays immediately after incubation (b, c) (*n* = 3). Representative morphology recorded with a microscope after treatment with GBCAs for 24 h (d).

**Figure 2:**
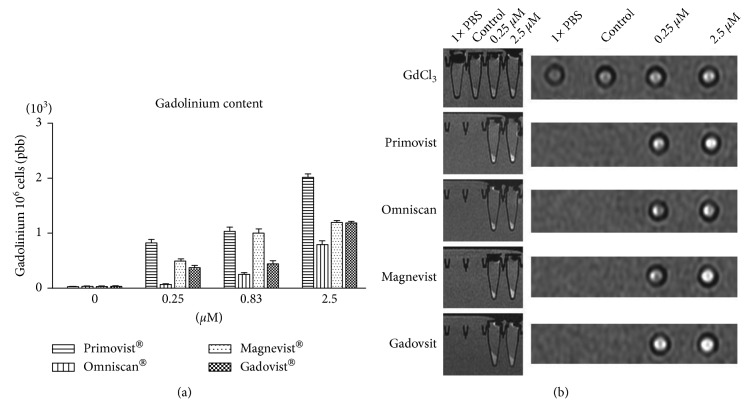
MRI and gadolinium content of macrophages treated with various gadolinium chloride and GBCAs. ICP-MS analysis showed that the RAW cells generally contained gadolinium after treatment with the various Gd chloride and GBCAs (a) (*n* = 3). Coronal view and T1-weighted scanning protocols were performed (b). The cells were centrifuged to the bottom of the test tube and imaged as dark signals. The signal intensities of the cells revealed a dose-dependent increase on treatment with higher concentrations of Gd chloride and GBCAs for 24 h.

We serially diluted Primovist®, Omniscan®, Magnevist®, and Gadovist® with PBS solution. Because different dissociation would result in different pH values, we tested the pH value of PBS diluted GBCAs. It is found that all of PBS diluted GBCAs kept neutral pH values (Figure 1(a)). Figures 1(b) and 1(c) show the viabilities of the cells of murine macrophage (RAW 264.7 cell line) stimulated by Gd chloride, Primovist®, Omniscan®, Magnevist®, and Gadovist® for 24 hours. No cell viability changes were observed with different kinds of GBCAs and Gd chloride (concentrations 0–2.5 *μ*M). No conformational changes in the cytology of RAW 264.7 were noted after 24 h of exposure to GBCAs. In the control and GBCAs stimulated cells in our experiment, the cell morphology generally showed a round form (Figure 1(d)). After 24 h incubation with 0–2.5 *μ*M GBCAs, the macrophages increased uptake of gadolinium as detected by inductively coupled plasma mass spectrometry (ICP-MS; Figure 2(a)). The incubation concentration of the GBCAs had a strong influence on the level of gadolinium uptake; the maximum gadolinium uptake was 2020.0 ± 47.6 ppb/10^6^ cells in the 2.5 *μ*M Primovist® treatment group, which was significant when compared to the control group. The presence of cellular GBCA uptake was also confirmed using cellular MRI, which demonstrated hyperintense dots at the bottom of the test tube. All of the cells, except those in the phosphate-buffered saline (PBS) treatment group, exhibited uptake of GBCAs regardless of the type of GBCAs used (Figure 2(b)).

### 3.2. ROS Production

**Figure 3:**
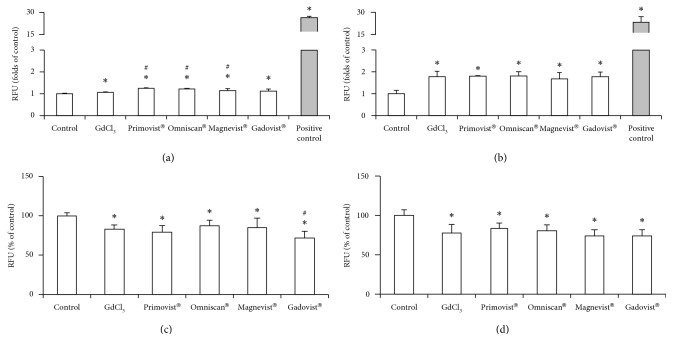
The effects of the different GBCAs and gadolinium chloride-induced reactive oxidative species and mitochondrial membrane potential in cultured RAW 264.7. The production of ROS was measured after treatment with 2.5 *μ*M Gd chloride or GBCAs for 4 h (a) and 24 h (b). Similarly, mitochondrial membrane potential was measured after 4 h (c) and 24 h (d) of treatment. *∗p* < 0.05 when compared with the control, #*p* < 0.05 when compared with Gd chloride. Positive control, H_2_O_2_ (2000 *μ*M) for 30 min.

We observed increased levels of ROS when the cells were exposed to either Gd chloride or GBCAs for 4 h. This effect was more significant in the 24 h exposure groups (Figures 3(a) and 3(b)). There was an almost two-fold increase in ROS levels in the Gd chloride and GBCAs treatment groups.

### 3.3. Decrease in Mitochondrial Membrane Potential

Treatment with Gd chloride or GBCAs resulted in a dramatic drop of mitochondrial membrane potential in macrophages at 4 and 24 h after induction (Figures 3(c) and 3(d)). Both Gd chloride and GBCAs demonstrated similar levels of mitochondrial membrane potential depression in macrophages. However, no statistically significant differences were observed between the 4 and 24 h treatment groups.

### 3.4. Effects on IL-6, IL-10, PGE2, Nitrite/Nitrate Production

**Figure 4:**
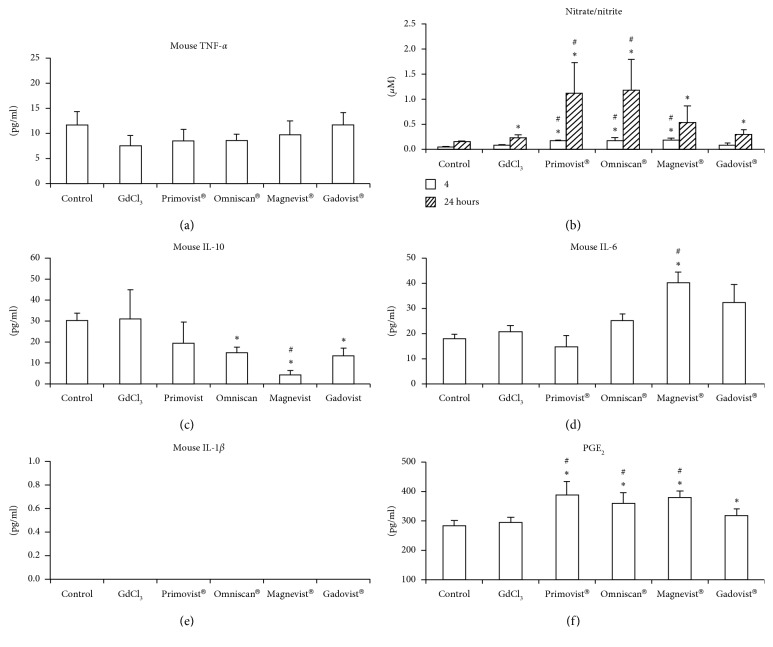
Effect of gadolinium or GBCAs on the production of TNF-*α*, nitrate/nitrite, IL-10, IL-1*β*, IL-6, and PGE2 by the RAW 264.7 cells. The levels of TNF-*α* (a) were measured after treatment with 2.5 *μ*M Gd chloride or GBCAs (2.5 *μ*M) for 4 h and those of nitrite/nitrate (b) for 4 h and 24 h IL-10 (c) for 4 h, IL-6 (d) for 24 h, IL-1*β* (e) for 24 h, and PGE2 (f) for 24 h were measured. *∗p* < 0.05 when compared with control. #*p* < 0.05 when compared with Gd chloride (*n* = 5).

We investigated the inflammatory effects of Gd^3+^ and GBCAs on macrophages (Figure 4). The administration of Gd^3+^ or GBCAs did not result in increasing the secretions of TNF-*α* and IL-1*β* in the 4 and 24 h groups (Figures 4(a) and 4(e)). Interestingly, macrophages treated with Magnevist® demonstrated a two-fold increase in IL-6 levels at 24 h (Figure 4(d)). Moreover, Omniscan®, Magnevist®, and Gadovist® inhibited IL-10 secretion in the same group (Figure 4(c)). Primovist®, Omniscan®, and Magnevist® increased nitrate/nitrite production at 4 and 24 h, and this effect was more significant in the 24 h group, except in the case of Magnevist® (Figure 4(b)). Furthermore, both Gd chloride and Gadovist® increased nitrate/nitrite production at 24 h; the increases were more noticeable in the Primovist®, and Omniscan® groups than in the Gd chloride group. Primovist®, Omniscan®, Magnevist®, and Gadovist® increased PGE2 production at 24 h (Figure 4(f)). The production of PGE2 in the Primovist®, Omniscan®, and Magnevist® groups were higher than that of the Gd chloride group at 24 h.

### 3.5. Effects on Cytokines Production after the Exposure of LPS

**Figure 5:**
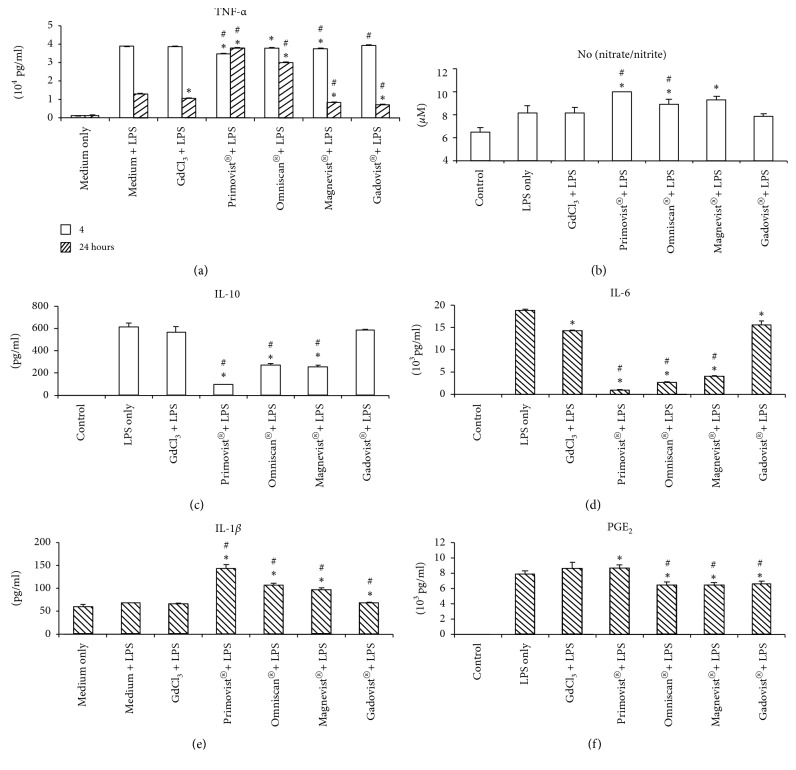
Effect of gadolinium or GBCAs on the production of TNF-*α*, nitrate/nitrite, IL-10, IL-1*β*, IL-6, and PGE2 by the RAW 264.7 cells after the stimulation of LPS. RAW 264.7 cells were treated 24 h with 2.5 *μ*M Gd chloride or GBCAs. The following cultures were stimulated for 4 or 24 h with 100 ng/ml or 1 *μ*M LPS. The levels of TNF-*α* (a) were measured after treatment for 4 h and 24 h, and those of nitrite/nitrate (b) for 4 h, IL-10 (c) for 4 h, IL-6 (d) for 24 h, IL-1*β* (e) for 24 h, and PGE2 (f) for 24 h were measured. *∗p* < 0.05 when compared with LPS only. #*p* < 0.05 when compared with Gd chloride and LPS exposure (*n* = 3).

Followed by overnight exposure of Gd chloride and GBCAs, macrophages were stimulated with 100 ng/ml or 1 *μ*g LPS for either 4 or 24 h. Then cytokine production was measured (Figure 5). In most cytokines secretion including TNF-*α* (4 h), nitrite/nitrate (4 h), and IL-10 (4 h), IL-1*β* (24 h), PGE2 (24 h) with a combination of Gd^3+^ and LPS is similar to the LPS treatment group. There is a decreasing secretion of TNF-*α* (24 h) and IL-6 (24 h) in Gd3^+^, in conjunction with the LPS group, when compared to the LPS stimulation group (Figures 5(a) and 5(d)). Primovist®, Omniscan®, and Magnevist® treatment resulted in a decreased secretion of TNF-*α* when compared to the LPS stimulation group at 4 h after LPS exposure (Figure 5(a)). Gd chloride, Magnevist®, and Gadovist® treatment resulted in a decreased TNF-*α* production when compared to the LPS stimulation group as well as the combination of LPS plus Gd chloride at 24 h. However, TNF-*α* secretion at 24 h post-LPS exposure was significantly increased in the presence of Primovist® and Omniscan®. The production of nitrate/nitrite and IL-1*β* was significantly increased in Primovist®, Omniscan®, and Magnevist® at 4 h or 24 h post-LPS exposure. The secretion of IL-10 or IL-6 decreased with the presence of Primovist®, Omniscan®, and Magnevist® at 4 h or 24 h post-LPS exposure (Figures 5(c), and 5(d)). The productions of PGE2 in the Omniscan®, Magnevist®, and Gadovist® groups were lower than both the LPS group and the combination of Gd chloride and LPS group, but the combination of Primovist®-LPS exposure increased at 24 h (Figure 5(f)).

## 4. Discussion

Our novel findings demonstrate that exposing to low concentration (2.5 *μ*M) of GBCAs can alter the immune function of macrophage regardless of the presence of LPS exposure. Despite that there is no obvious GBCA-mediated cytological changes, we observed different gadolinium concentrations in macrophages measured by ICP-MS. However, the actual subcellular compartment in which gadolinium accumulates needs further investigation. The accumulation of gadolinium was most pronounced in the Primovist®-treated group. It is known that organic anion transporting polypeptide (OATP) is responsible for transferring Primovist® into the cytoplasm, and cancer cell lined with overexpressed OATP has higher intracellular Primovist® deposition [16, 19]. The expression of OATP in the macrophage is not fully determined, but it is an evident route for certain kinds of GBCAs to go into the intracellular space. Future studies to investigate OATP expression levels in macrophages and their potential effects following the Primovist® exposure are clinically relevant.

### 4.1. Exposure to GBCAs Increases Oxidative Stress

In our present study, Gd chloride and GBCAs stimulated the production of ROS and suppressed the potential of the mitochondrial membrane. These effects were observed at a clinical practice concentration. Both the increase of ROS and the decrease of mitochondrial membrane potential have been reported in some studies related to environmental hazard toxicity, particularly those induced by heavy metals [20, 21]. Mitochondria have been suggested to be both the source and target of ROS [22]. Abnormal accumulation of ROS in cells can trigger downstream events of apoptosis and cytokine release [23, 24]. Low levels of GBCAs exposure increased oxidative stress in murine macrophages in the current study. This finding might have some impacts in several pathological conditions such as NSF because previous studies showed that the increase of ROS is related to this disease [25].

### 4.2. GBCA Exposure Induces Nitric Oxide and Prostaglandin E2 Production

Our study showed increased PGE2 secretion in all macrophages after 24 h exposure to GBCAs. Nitrate/nitrite levels originating from the murine macrophages were also elevated in the Primovist®, Omniscan®, and Magnevist® groups after 4 and 24 h of exposure. Both nitrate/nitrite and PGE2 are considered as inflammatory and immunomodulatory mediators in the mammalian physiology [26]. They also play a major role in chemical carcinogenesis [27]. Several oxidative stressors can induce the expression of iNOS and COX-2, which synthesize NO and PGE2, respectively [28]. Some studies have indicated that iNOS and COX-2 expression pathways are induced in vivo by models involving both inflammatory and oxidative stress conditions [29]. Further in vivo studies to verify the effects of GBCAs at low concentrations are needed.

### 4.3. Cytokines Secretion after the Combination of GBCAs and LPS Exposure

We observed differences in cytokine profiles in response to GBCAs and LPS exposure compared to LPS only. Primovist®, Omniscan®, and Magnevist® upregulated the expression of nitrate/nitrite at 4 h, IL-1*β* at 24 h and TNF-*α* at 24 h in LPS-stimulated macrophages. They also inhibited the expressions of anti-inflammatory molecules of IL-10 at 4 h and inflammatory cytokines of IL-6 at 24 h. TNF-*α* is an early stage cytokine after the LPS stimulation [30]. However, the levels of TNF-*α* were still high even after LPS exposure for 24 h. This scenario should be studied in the future. Most cytokines secretion in our studies such as TNF-*α*, nitrite/nitrate, IL-10, and IL-1*β* after the stimulation of Gd chloride, Gadovist®, and LPS exposure was similar to the LPS exposure except IL-6 and PGE2. The combination of Primovist®, Omniscan®, and Magnevist® and LPS exposure impaired the immune responses. The impaired levels of cytokines with this Gadovist® were the least significant when compared to the other three GBCAs in this study.

### 4.4. The Relationship between the Chemical Structure of GBCAs and Inflammatory Changes in the Macrophage

The current study indicated that Gadovist® was the least toxic GBCA. Although the maximum and minimum gadolinium cellular uptake concentrations were observed with Primovist® and Omniscan®, respectively, the uptake was not correlated with the toxicity of the GBCAs. This may be attributed to several reasons. Macrocyclic GBCAs, such as Gadovist® or Dotaram, exhibited lower dissociation constants, and the molecular structure of macrocyclic GBCAs is more stable than the linear GBCAs [31]. Among the four GBCAs investigated in the current study, Gadovist® was a macrocyclic GBCAs, whereas the remaining three were linear agents. Macrocyclic GBCAs form ring-shaped structures with Gd^3+^ surrounded by an organic chelating portion, making it harder for gadolinium to dissociate from this encircled chelating environment. The higher the dissociation constant is, the freer gadolinium can be released into the circulation and tissues [32]. The gadolinium released from the chelating complex induces the activation of various profibrotic molecular pathways in one or more of the cell types existed in fibrotic NSF lesions, such as macrophages, fibroblasts, and fibrocytes [33]. A previous study reported that according to the chelate model of gadolinium, the entire Gd^3+^ chelating complex, not just the transmetallated gadolinium, was involved in the pathophysiology of NSF [34]. In the present study, the macrophages released some cytokines that may be related to NSF even at very low GBCA concentrations. We also compared the cellular responses toward free and chelated gadolinium. However, when Gd chloride is added in DMEM which contains phosphate, the formation of insoluble Gd phosphate is unavoidable. We cannot identify the true toxicity of gadolinium because the proper amount of gadolinium uptake by the macrophages is unpredictable. The toxicity of gadolinium is likely to be underestimated in our study. Although we noticed the neutral pH of the PBS diluted solution after 24 h incubated with macrophage in all contrast medium groups, the conditions do not mimic the intracellular and intralysosomal pH that likely exists in vivo. The concern of the existence of dechelation of gadolinium after encountering acidic solution may come from macrophage metabolism. Further studies are required to differentiate between the toxic levels of GBCAs originating from dissociated gadolinium and those from chelated Gd^3+^ complexes.

Macrocyclic Gadovist® elicited lower immune responses from macrophages with marginal ROS elevation and potential mitochondrial membrane suppression. No nitrate/nitrite stimulation was observed after 4 h exposure to Gadovist®. Moreover, the levels of cytokines under LPS exposure were similar to the combination of the LPS group and were the least significant when compared with the other three GBCAs in this study. These findings may be attributed to the macrocyclic chemical nature of this GBCA, which makes it less influential on the macrophage functions.

Previous studies have reported that ROS and cytokines were induced after incubation with high concentrations (0.5–10 mM) of GBCAs in monocytes [13, 14]. The human study has revealed maximum plasma gadolinium concentrations of 65.7 *μ*g/mL [15]. We assume that the gadolinium concentrations releasing from gadolinium retention tissue are far less than 65.7 *μ*g/mL. In the current study, we focused on a lower concentration (2.5 *μ*M) of GBCAs to simulate the real clinical conditions. Interestingly, our results showed that toxicities existed despite of the low concentration of GBCAs used.

Although a variety of cytokines were released from macrophages cultured with different kinds of GBCAs, we acknowledge that the increase in ROS, IL-6, nitrite/nitrate, and PGE2 levels and the decrease in mitochondrial membrane potential induced by GBCAs in the current study are not analyzed in the clinical practice. We think macrophage morphology is one of the critical issues in the research of cytokine release. When stimulated, the cells become stellate-like that is a good indicator for determination of strong irritation. In the control and GBCAs stimulated cells in our experiment, the cell morphology generally showed no conformational changes. Our results showed no strong irritation from the GBCAs that is compatible with our cytokine analysis. With the increasing reports on the deposition of gadolinium in brain, bone, and renal tissues of patients with normal renal function exposed to GBCAs during MRI examinations [35], the potential toxic effects of low level GBCAs in various tissues must be investigated thoroughly. The accumulated GBCAs in the human body may stimulate macrophage and alter the immune reaction of macrophages after LPS stimulation. Our data showed that low levels of GBCAs could induce a potent activation of the macrophages and suggested a possible mechanism that may be related to the potential toxicity of GBCAs.

To the best of our knowledge, this is the first study that focuses on the effects of low concentrations (2.5 *μ*M) of GBCAs on macrophage responses. GBCAs exerted a variety of impacts on the macrophages even at low concentrations, indicating that it is capable of inducing several pathophysiological events that might be related to NSF or the accumulation of gadolinium in different tissues. Further in vitro and in vivo studies to evaluate the effects of low levels of GBCAs on immune cell response should be conducted.

In conclusion, similar to physiological conditions, exposing to low levels of GCBAs can also alter the macrophage function and elicited a variety of immune responses in murine macrophages in our present study. Further investigation of GBCAs on the human macrophage or *in vivo* animal study may clarify the role of macrophage on the pathogenesis of NSF and other GBCAs-related disease.

## Data Availability

The data used to support the findings of this study are included within the article.

## Supplementary Materials

Supplementary Materials Supplement 1. The effects of the very low doses of GBCAs and gadolinium chloride-induced reactive oxidative species and mitochondrial membrane potential in cultured RAW 264.7 were observed. The production of ROS was measured after treatment with 0.25 and 0.83 *μ*M Gd chloride or GBCAs for 4 h (A: 0.25 *μ*M, B: 0.83 *μ*M) and 24 h (C: 0.25 *μ*M, D: 0.83 *μ*M). Similarly, mitochondrial membrane potential was measured after 4 h (E: 0.25 *μ*M, F: 0.83 *μ*M) and 24 h (G:0.25 *μ*M, H: 0.83 *μ*M) of treatment. H2O2 (2000 *μ*M) for 30 min (*n* = 3). Supplement 2. The effect of gadolinium or GBCAs on the production of nitrate/nitrite (24 h), IL-1*β* (4 h), and IL-6 (4 h) by the RAW 264.7 cells after the stimulation of LPS was observed. RAW 264.7 cells were treated 24 h with 2.5 *μ*M Gd chloride or GBCAs. The following cultures were stimulated for 4 or 24 h with 100 ng/ml or 1 *μ*M LPS. The levels of nitrate/nitrite (A) were measured after treatment for 24 h, and IL-1*β* (B) and IL-6 (C) for 4 h for 24 h were measured. *∗p* < 0.05 when compared with LPS only. #*p* < 0.05 when compared with Gd chloride and LPS exposure (*n* = 3).
