# Supplementary material for: Exposure of Macrophages to Low-Dose Gadolinium-Based Contrast Medium: Impact on Oxidative Stress and Cytokines Production
Source: Contrast Media Mol Imaging. 2018 Dec 2;2018:3535769. doi: 10.1155/2018/3535769 (PMC6305030; doi:10.1155/2018/3535769)

**Supplement 1. The effects of the very low doses of GBCAs and Gadolinium Chloride-induced reactive oxidative species and mitochondrial membrane potential in cultured RAW264.7.**

The production of ROS was measured after treatment with0.25 and 0.83μM Gd chloride or GBCAs for 4 h (A:0.25μM, B: 0.83μM) and 24 h (C:0.25μM, D:0.83μM). Similarly, mitochondrial membrane potential was measured after 4 h ( E: 0.25μM, F:0.83μM) and 24 h (G:0.25μM, H:0.83μM) of treatment. H2O2 (2000 μM) for 30 min.(n=3)

**Supplement 2. Effect of gadolinium or GBCAs on the production of nitrate/nitrite (24h), IL-1β(4h) and IL-6 (4h), by the RAW 264.7 cells after the stimulation of LPS.**

RAW 264.7 cells were treated 24h with 2.5μM Gd chloride or GBCAs. The following cultures were stimulated for 4 or 24 h with 100 ng/ml or 1μM LPS. The levels of nitrate/nitrite (A) were measured after treatment for 24h, and IL-1β (B) and IL-6 (C) for 4h for 24 h were measured . *p < 0.05 when compared with LPS only. # p < 0.05 when compared with Gd chloride and LPS exposure. (n = 3).


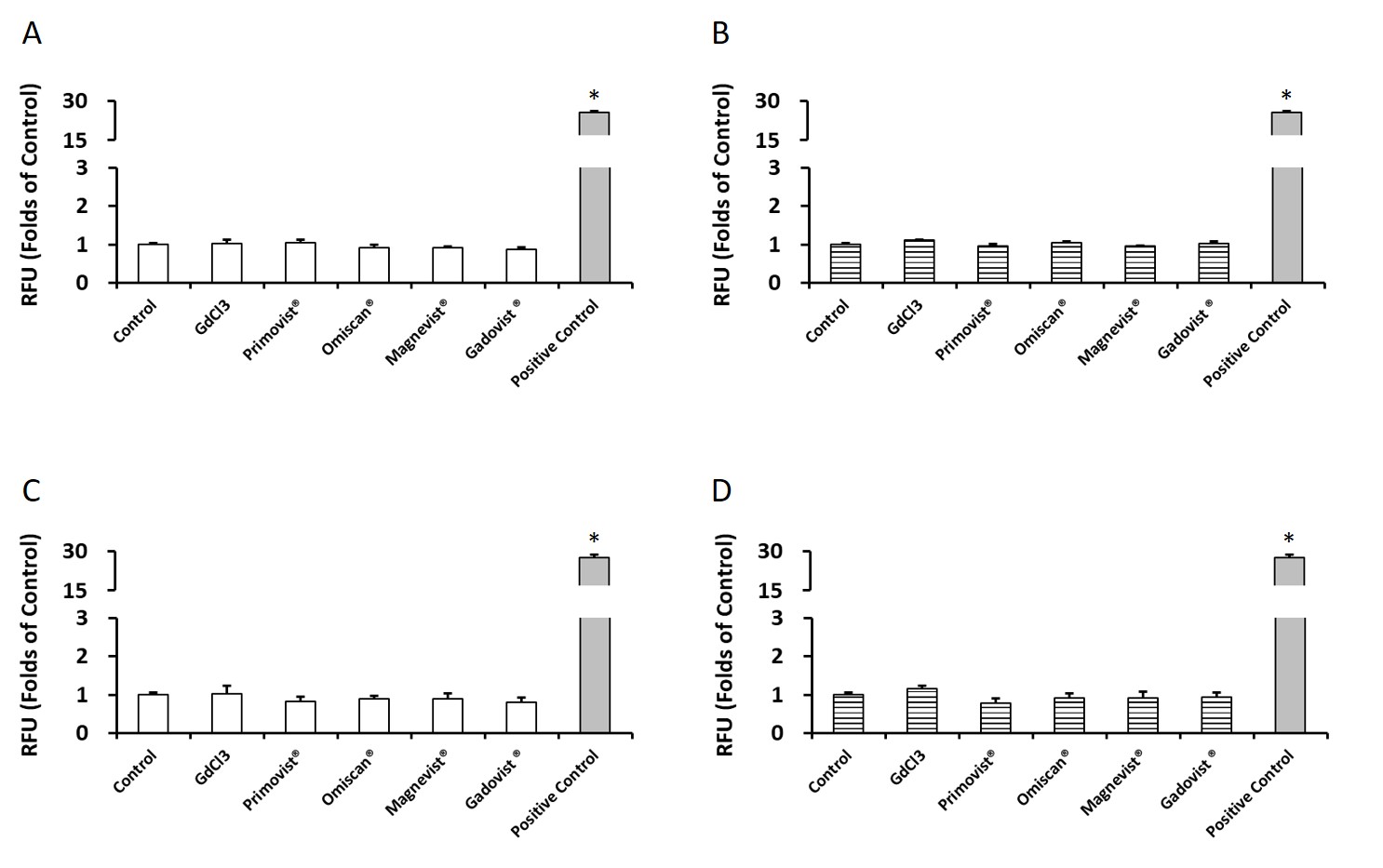
Supplement 1


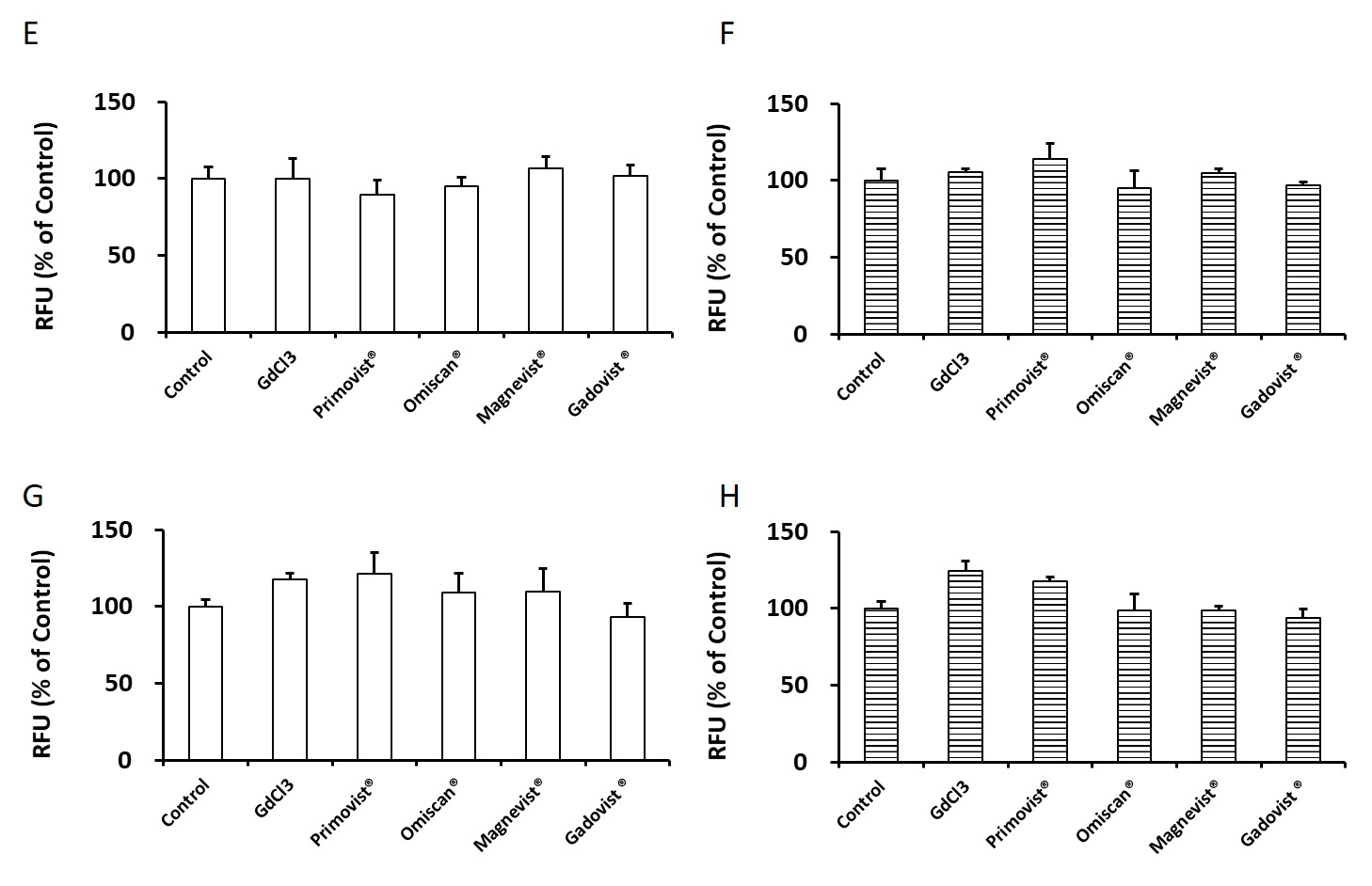


Supplement 2


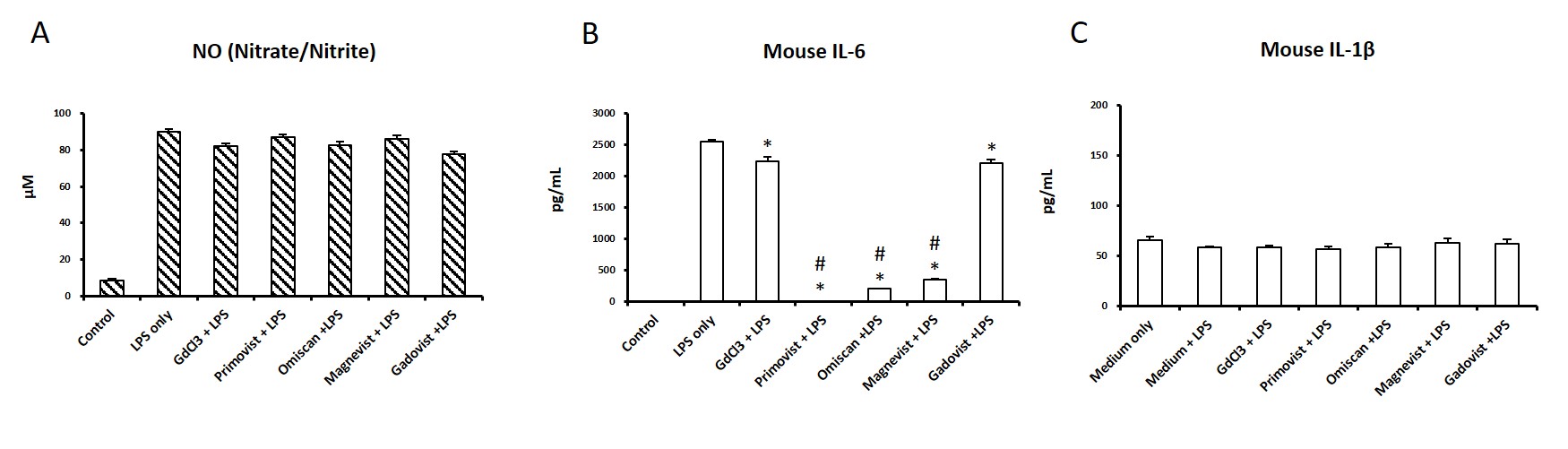

Supplement: Supplementary Materials — Supplement 1. The effects of the very low doses of GBCAs and gadolinium chloride-induced reactive oxidative species and mitochondrial membrane potential in cultured RAW 264.7 were observed. The production of ROS was measured after treatment with 0.25 and 0.83 μM Gd chloride or GBCAs for 4 h (A: 0.25 μM, B: 0.83 μM) and 24 h (C: 0.25 μM, D: 0.83 μM). Similarly, mitochondrial membrane potential was measured after 4 h (E: 0.25 μM, F: 0.83 μM) and 24 h (G:0.25 μM, H: 0.83 μM) of treatment. H2O2 (2000 μM) for 30 min (n = 3). Supplement 2. The effect of gadolinium or GBCAs on the production of nitrate/nitrite (24 h), IL-1β (4 h), and IL-6 (4 h) by the RAW 264.7 cells after the stimulation of LPS was observed. RAW 264.7 cells were treated 24 h with 2.5 μM Gd chloride or GBCAs. The following cultures were stimulated for 4 or 24 h with 100 ng/ml or 1 μM LPS. The levels of nitrate/nitrite (A) were measured after treatment for 24 h, and IL-1β (B) and IL-6 (C) for 4 h for 24 h were measured. ∗p < 0.05 when compared with LPS only. #p < 0.05 when compared with Gd chloride and LPS exposure (n = 3). [file 3535769.f1.docx]
